# Frequency, severity, and factors associated with clinically significant drug-drug interactions among patients with cancer attending Mbarara Regional Referral Hospital Cancer Unit, Uganda

**DOI:** 10.1186/s12885-022-10396-8

**Published:** 2022-12-05

**Authors:** Bonny Luzze, Barnabas Atwiine, Henry Mark Lugobe, Tadele Mekuriya Yadesa

**Affiliations:** 1 Department of Pharmacy, Mbarara University of Science and Technology, Mbarara, Uganda; 2 Department of Pediatrics and Child Health, Mbarara University of Science and Technology, Mbarara, Uganda; 3 Cancer Unit, Mbarara Regional Referral Hospital, Mbarara, Uganda; 4 Department of Obstetrics and Gynecology, Mbarara University of Science and Technology, Mbarara, Uganda; 5 Pharm-Biotechnology and Traditional Medicine Center, Mbarara University of Science and Technology, Mbarara, Uganda; 6 Department of Pharmacy, Ambo University, Ambo, Ethiopia

**Keywords:** Frequency, Factors associated, Drug-drug interactions, Cancer

## Abstract

**Background:**

Cancer is a major public health problem with pharmacotherapy being the cornerstone of its management. Cancer patients receive multiple drugs concurrently risking Drug-Drug Interactions (DDIs). DDIs, though avoidable, can significantly contribute to morbidity, mortality, and increased healthcare costs in this population of patients. Currently, there is no published study from Uganda on clinically significant DDIs (cs-DDIs) among cancer patients. This study identifies frequency, severity, and factors associated with cs-DDIs at Mbarara Regional Referral Hospital Cancer Unit (MRRHCU).

**Method:**

A cross-sectional study was conducted among 300 cancer patients receiving chemotherapy from a tertiary care hospital in western Uganda from January–February 2022. A questionnaire and data collection form were used to collect patient data. Lexicomp® Drug interaction software was used to screen the patient drug information for DDIs and assess their severity. Predictors of DDIs were identified using logistic regression using SPSS (Statistical Package for Social Sciences).

**Result:**

Three hundred participants were enrolled with a mean age of 48 ± 23.3 years. One hundred eighty-one patients experienced 495 cs-DDIs; with a mean of 1.7 ± 2.2. The prevalence of cs-DDI was 60.3% (55.0-66.0% at 95% CI). Digestive organ neoplasms were the most commonly (80, 26.7%) diagnosed category, and ‘plant alkaloids and other natural products were the most frequently (143, 47.7%) used chemotherapeutic drug classes. About three-quarters of cs-DDIs were rated as category C risk (367, 74.1%) whereas over two-thirds (355, 71.7%) were moderate in severity.. Being female (aOR = 2.43 [1.23–4.48 at 95% CI]; *P*-value = 0.011) and use of ≥ 6 drugs concurrently (aOR = 18.82 [9.58–36.95 at 95% CI]; *P*-value < 0.001)) were significantly associated with cs-DDIs.

**Conclusion:**

More than half of the participants experienced at-least one cs-DDI which is generally higher than what was reported in high-income settings. About three-quarters were category C and moderate in severity, and require enhanced monitoring for safety and treatment outcome. Being female and using ≥ 6 drugs were significantly associated with cs-DDIs.

## Introduction

Drug interaction is defined as a change in the effect of a drug as a result of its interaction with another drug(s), food, or disease [1]. The types of drug interactions include drug-drug interactions (DDIs), food-drug interactions, disease-drug interactions, and drug-supplement interactions. DDIs are the commonest, attributing approximately 20% to 30% of all adverse events (AEs) [1]. DDIs may cause a reduction in therapeutic efficacy [2] or unexpected adverse effects [3, 4] negatively affecting treatment outcomes. The drugs involved can be prescription-only medicines or over-the-counter medicines. DDIs can be classified as pharmacokinetic or pharmacodynamic in nature [5, 6], with pharmacokinetic DDIs being the commonest [7]. A systemic review and meta-analysis approximated that 1/10 hospitalized patients are exposed to cs-DDIs [2], 20–40% [5]. Studies have shown that DDIs have become prevalent among cancer patients, which may be due to disease sequelae that require pharmacologic management, hence contributing to polypharmacy which is one of the leading DDI risk factors. The factors associated with DDIs include lack of DDI identification causality tools and DDI lists [2], long hospital stay, polypharmacy particularly among the elderly because of comorbidities and complex therapy regimens [5, 8], increased approval of combination drug formulations some of which exhibit highly complex drug interaction profiles [9] and self-medication [10] have contributed greatly to the burden.

Among ambulatory cancer patients, 27–58% of them are at risk of DDIs involving anticancer agents like doxorubicin, cyclophosphamide, and methotrexate among others, with methotrexate being the most implicated agent [11, 12]. In Uganda, a retrospective analysis showed a 23% prevalence of potential DDIs in only the major wards (medical, surgical, obstetrics/gynecology, and pediatrics) [13] excluding the cancer unit. In Kenya, a study among cervical cancer patients identified a 46.9% prevalence of DDIs [14]. Generally, in East Africa, DDIs have not been studied exclusively in the cancer units. Previous studies emphasized specific types of drug interactions like herbal-drug interactions (NDA, 2018), and other specific diseases like HIV (89.3% prevalence of potential DDIs) [15] and malaria [16] among others. DDI databases with a warning system combined with a pharmacist’s assessment can be used to identify, prevent, and resolve DDIs [2]. In Uganda, according to published data, there was no study done about DDIs, specifically among cancer patients on chemotherapy. This study aimed to determine the frequency, severity, and factors associated with cs-DDIs in cancer patients at MRRHCU.

## Methods

### Study design and setting

A cross-sectional study was carried out at the MRRHCU, Southwestern Uganda (269.7 km from the capital city Kampala), a public sector tertiary care teaching hospital from January to February 2022. The cancer unit has a bed capacity of 20 and 18 beds for adults and pediatric patients respectively; with two oncology specialists, one pharmacist, and seven nurses. At the study site, there was neither an active screening system for DDIs nor standardized tools for DDI detection and prevention.

### Study participants and selection criteria

All adult and pediatric patients with cancer that received chemotherapy from January–February 2022 at MRRHCU were considered in the current study. The study included all patients with cancer of either sex who had a confirmed cancer diagnosis who gave their written consent to participate in the study and who were prescribed and receiving at least one chemotherapy drug. Whereas patients who withdrew from the study before completion of enrollment and those receiving only one drug were excluded from the study.

### Data collection and screening/assessing DDIs

A questionnaire and data collection form were developed, validated, and used to document patients’ baseline socio-demographics, past medical history, and drug use from each eligible patient and their medication file during their attendance at the unit. Both English and Runyankole questionnaires were developed and pre-tested with 15 patients (5% of the estimated sample size) at MRRHCU before actual data collection to ensure the feasibility of the data collection tool. Results from the pretest were excluded from the final analysis. The chemotherapeutic agents and any other concurrent medications prescribed were recorded in the data collection form, by the principal investigator assisted by the assistants.

Two research assistants, a nurse, and a medical laboratory scientist, both bachelor's degree holders were trained on the data collection protocols and ethical considerations before the study started to ensure data quality. Participants voluntarily consented before enrolment after their clinical review by the clinical team. All medications to be taken by the patient were recorded by the research assistants and the principal investigator through direct interviews and patients’ medical records review using a data collection tool on recruitment into the study.

Upon getting the list of drugs the patient was taking, the drugs were entered into the Lexicomp® drug interaction software for potential DDI screening and assessment on risk category and severity. The software assigned a risk rating of A (no known interaction), B (no action needed), C (monitor therapy), D (consider therapy modification), and X (avoid combination). Combinations rated C, D and X indicated cs-DDIs that needed the clinician’s attention while A and B were less clinical. It assigned major, moderate, or minor in severity terms. To minimize the possible consequent bias, the identified cs-DDIs were not intervened except for those believed by investigators to be potentially life-threatening, and these recommendations were made during ward rounds without letting the clinical team know it was from the study’s findings.

### Data processing and analysis

Statistical Package for Social Sciences (SPSS), version 21, a software program, was used for data management and quantitative analysis (logistic regression, descriptive, and factor analysis). A descriptive analysis of socio-demographic, clinical, and drug-related variables was presented using a mean with an interquartile range and percentages.

The frequency of cs-DDIs among participants was calculated by dividing the number of participants with at least one cs-DDIs by the total number of participants and expressed as a percentage. Univariate and multivariate logistic regression was employed to determine independent factors associated with cs-DDIs. Variables with *p* < 0.25 at univariate analysis were included in the multivariate logistic regression. In the multivariate model, *P*-values < 0.05 were considered statistically significant.

## Results

### Participant characteristics

Three hundred fifteen patients were approached of whom 10 were on follow-up on complete remission after chemotherapy, 2 declined to receive treatment after review, and 3 declined to consent to participate. In total, 300 patients were studied including 239(79.9%) outpatients, the majority (250, 83.3%) from the adult ward. Over half (163, 54.3%) were males whereas the majority (184, 61.33%) were below 60 years of age and the mean age was 48.0 ± 23.3. Over half (167, 55.7%) of the participants had a previous admission (Table 1).

**Table 1:** The socio-demographic characteristics of cancer patientsUganda

Variables	Category	Frequency	Percentage (%)
Patient's gender	Female	137	45.7
	Male	163	54.3
Age Mean ± SD (48.0 ± 23.3)	< 18	51	17.0
	18–59	133	44.3
	> = 60	116	38.7
Ward setting	Adult ward	250	83.3
	Pediatrics	50	16.7
Marital status (N = 250)	Married	199	79.6
	Single	24	9.6
	Separated/Divorced/Widowed	27	10.8
Place of residence	Urban	57	19.0
	Rural	243	81.0
Patient's employment status (*N* = 250)	Unemployed	40	16.0
	Employed	210	84.0
Level of education	None	83	29.4
	Primary	137	48.6
	Secondary/Tertiary	62	22.0
Patient's hospital attendance status	In Patient	61	20.3
	Out Patient	239	79.7
Previous admission	No	167	55.7
	Yes	133	44.3
Previous or concurrent radiation/surgery	No	215	71.7
	Yes	85	28.3

### Types of cancers documented

**Fig. 1:**
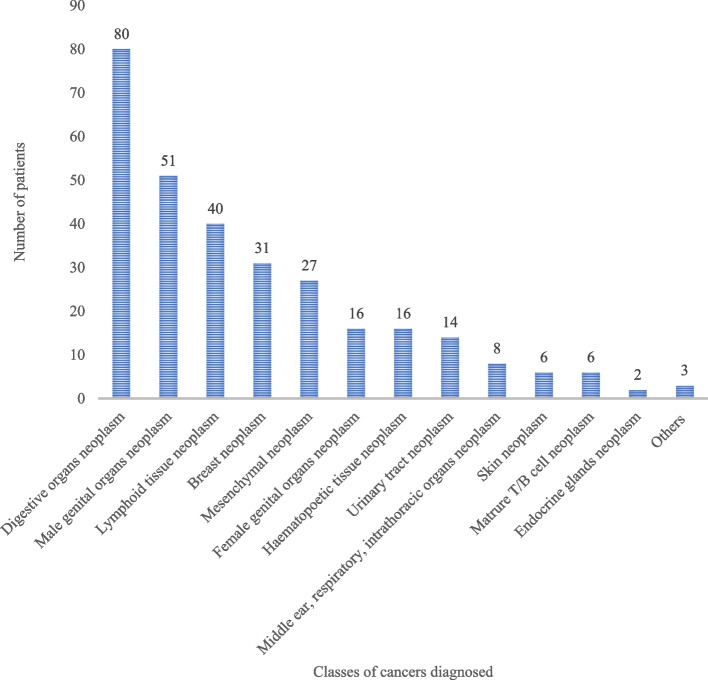
The types of cancers documented among patients at the cancer unit, Uganda. Others: Kaposi sarcoma, peripheral nerve sheath tumor, neck tumor

Digestive organs neoplasms (80, 26.7%) followed by male genital organs neoplasms (51, 17.0%), and lymphoid tissue neoplasms (40, 13.3%) were the most neoplasms diagnosed (Fig. 1).

### Chemotherapeutic agents used

The study participants used a mean of 1.87 ± 0.82 chemotherapeutic drugs. Out of the 8 classes of chemotherapeutic drugs, ‘plant alkaloids and other natural products were used by almost half (143, 47.7%) of the patients followed by ‘antimetabolites’ used by 103 (34.3%). vincristine (57, 19%), docetaxel (44, 14.7%), doxorubicin (44, 14.7%), cyclophosphamide (41, 13.7%), and goserelin (35, 11.7%) were the top five most commonly used specific chemotherapeutic agents (Table 2).

**Table 2:** The classes and specific chemotherapeutic agents used by cancer patients, Uganda

Classes of chemotherapeutic agents (Frequency, Percentage)	Specific chemotherapeutic agents (Frequency, Percentage)
Plant alkaloids and other natural products (143, 47.7%)	Vincristine (57, 19%), Docetaxel (44, 14.7%), Paclitaxel (29, 9.7%), Etoposide (13, 4.3%), Irinotecan (2, 0.7%), Vinblastine (2, 0.7%)
Antimetabolites (103, 34.3%)	Capecitabine (32, 10.7%), Fluorouracil (30, 10%), Methotrexate (30, 10%), Gemcitabine (14, 4.7%), Mercaptopurine (10, 3.3%), Cytarabine (10, 3.3%)
Platinum analogues (66, 22%)	Oxaliplatin (32, 10.7%), Cisplatin (25, 8.3%), Carboplatin (9, 3%)
Cytotoxic antimetabolites (62, 20.7%)	Doxorubicin (44, 14.7%), Bleomycin (10, 3.3%), Epirubicin (7, 2.3%), Dactinomycin (7, 2.3%), Daunorubicin (5, 1.7%)
Alkylating agents (58, 19.3%)	Cyclophosphamide (41, 13.7%), Dacarbazine (9, 3%), Ifosfamide (3, 1%), Melphalan (3, 1%), Chlorambucil (2, 0.7%)
Endocrine therapy (53, 17.7%)	Goserelin (35, 11.7%), Bicalutamide (27, 9%), Tamoxifen (6, 2%), Abiraterone (4, 1.3%), Anastrozole (2, 0.7%)
Protein kinase inhibitors (23, 7.7%)	Imatinib (15, 5%), Erlotinib (6, 2%), Nintedanib (2, 0.7%), Sorafenib (1, 0.3%), Bortezomib (1, 0.3%)
Miscellaneous (14, 4.7%)	Thalidomide (6, 2%), Asparaginase (5, 1.7%), Arsenic-trioxide (2, 0.7%), Rituximab (1, 0.3%), Tamsulosin (1, 0.3%)

### Frequency of cs-DDIs

Out of 300 participants, 181 experienced at least one cs-DDIs giving a 60.3% (55.0%-66.0% at 95% CI) frequency. 495 cs-DDIs with a mean of 1.7 ± 2.2 were identified.

### Risk category and severity of the identified cs-DDIs

**Fig. 2:**
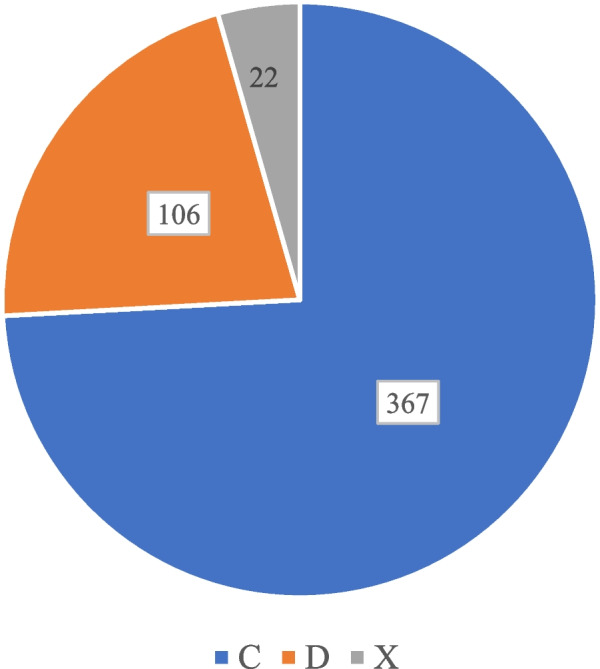
The risk categories of clinically significant drug-drug interaction among cancer patients at the cancer unit, Uganda. c- Risk rating that requires therapy monitoring; d – Risk rating that requires therapy modification; x- Risk rating that requires therapy avoidance

**Fig. 3:**
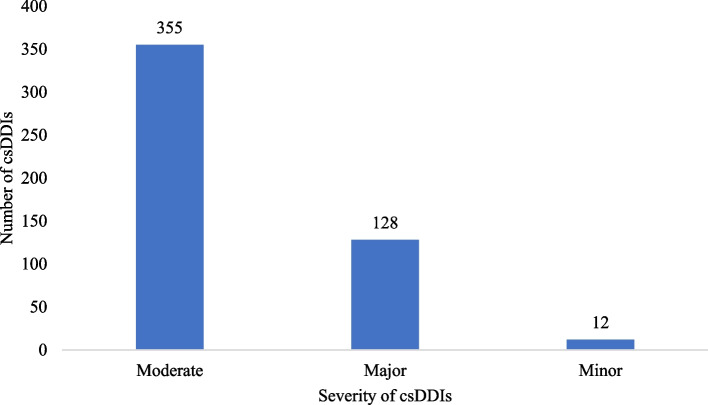
The Severity of clinically significant drug-drug interaction among cancer patients at the cancer unit, Uganda

Of the 495 cs-DDIs identified, 74.1% (367) were category C (Fig. 2). In severity, minor, moderate, and severe DDIs were 12 (2.4%), 71.7% (355), and 25.9% (128) respectively (Fig. 3).

### Frequency of cs-DDIs across classes of cancers

Endocrine gland neoplasms (100%), skin neoplasms (100%), and female genital organs neoplasms (87.5%) were associated with the highest frequency of cs-DDIs (Table 3).

**Table 3:** Prevalence of clinically significant DDIs among the different cancer classes diagnosed among cancer patients Uganda

Classes of cancers	Frequency of DDIs	Prevalence of cs-DDIs (%)
Urinary tract neoplasm	1	7.1
Male genital organs neoplasm	19	37.3
Female genital organs neoplasm	14	87.5
Breast neoplasm	21	67.7
Digestive organs neoplasm	60	75.0
Mesenchymal neoplasm	12	44.4
Lymphoid tissue neoplasm	27	67.5
Haematopoetic tissue neoplasm	8	50.0
Middle ear,respiratory, intrathoracic organs neoplasm	6	75.0
Skin neoplasm	6	100.0
Mature T/B cell neoplasm	3	50.0
Endocrine gland neoplasm	2	100.0
Others	2	66.7

*Others:* Kaposi sarcoma, peripheral nerve sheath tumor, neck tumor

### Drugs implicated in cs-DDIs

Doxorubicin (33, 6.7%), cyclophosphamide (33, 6.7%), and methotrexate (30, 6.1%) were the specific chemotherapeutic agents mostly involved in the cs-DDIs. On the other hand, morphine (103, 21.0%), metoclopramide (92, 19.0%), and dexamethasone (54, 11.0%) were the pre-medications mostly implicated in the cs-DDIs (Table 4). Morphine-metoclopramide was the commonest (*n* = 48) cs-DDI. Among the anti-cancers, cyclophosphamide-doxorubicin contributed to the highest cs-DDI (*n* = 28) followed by taxanes-platinum analogues (*n* = 18); both of which are major in severity. Among the other medications, morphine-metoclopramide followed by dexamethasone-NSAIDS (*n* = 14) were the most frequent (Table 5).

**Table 4:** The specific chemotherapeutic agents and premedications implicated in clinically significant Drug-drug interactions among cancer patients, Uganda

Drug	Frequency	Percentage (%) *N* = 495
**Chemotherapeutic agents**		
Doxorubicin	33	6.7
Cyclophosphamide	33	6.7
Methotrexate	30	6.1
Paclitaxel	21	4.2
Fluorouracil	17	3.4
Capecitabine	17	3.4
Docetaxel	13	2.6
Mercaptopurine	12	2.4
Thalidomide	9	1.8
Oxaliplatin	7	1.4
Cisplatin	7	1.4
Carboplatin	5	0.8
Goserelin	5	0.8
Imatinib	4	0.8
Erlotinib	4	0.8
Bortezomib	3	0.6
Bicalutamide	1	0.2
**Premedications**^a^**/Other drugs**		
Morphine	103	21
Metoclopramide	92	19
Dexamethasone	54	11
Ondansetron	51	10
Fosaprepitant	11	2.2

^a^The administered drugs minutes to hours before cancer chemotherapy to prevent adverse events such as hypersensitivity or nausea and vomiting due to cancer chemotherapy

**Table 5:** The most frequent cs-DDIs among cancer patients, Uganda

cs-DDI	Frequency	Severity (category)	Evidence	Potential Adverse outcome
Morphine + Metoclopramide	48	Moderate (C)	Fair	Drowsiness, somnolence (due to CNS depression enhancement by Metoclopramide)
Cyclophosphamide + Doxorubicin	28	Major (C)	Fair	Increased risk of cardiotoxicity
Taxanes + Platinum Analogues	18	Major (D)	Fair	Enhanced myelosuppression
Dexamethasone + NSAIDS	14	Moderate (C)	Fair	Enhanced NSAID adverse effects (bleeding)
Methotrexate + Proton Pump Inhibitors	13	Moderate (D)	Fair	Mucositis, Myalgias due to Methotrexate toxicity
Fluorouracil + Folinic Acid	12	Major (C)	Fair	Increased concentrations of fluorouracil (fluorouracil toxicity (granulocytopenia, anaemia, thrombocytopenia, stomatitis, vomiting)
Ondansetron + Domperidone	11	Moderate (D)	Fair	Increased risk of QT interval prolongation
Capecitabine + Proton Pump Inhibitors	11	Moderate (C)	Fair	Decreased capecitabine effect
Methotrexate + Mercaptopurine	10	Moderate (C)	Fair	Increased serum concentration of Mercaptopurine
Dexamethasone + Antihyperglycemic agents	10	Moderate (C)	Fair	Hyperglycemia (decrease antihyperglycemic agents’ effect)
Taxanes + Antihypertensives	9	Moderate (C)	Fair	Hypotension
Morphine + Antihypertensives	9	Moderate (C)	Fair	Hypotension (enhanced hypotensive effect of morphine)
Dexamethasone + Quinolones	9	Moderate (C)	Good	Increased risk for tendonitis and tendon rupture
Dexamethasone + Fosaprepitant	9	Minor (D)	Good	Gastric bleeding (increased dexamethasone serum concentration)
Ondansetron + Quinolones	8	Moderate (C)	Fair	Increased risk of QT interval prolongation
Antihistamines + Metoclopramide	7	Moderate (C)	Fair	Drowsiness, somnolence (due to CNS depression enhancement by Metoclopramide)

### Factors associated with cs-DDIs

Seven variables including age (COR = 2.36 [1.22–4.55 at 95% CI]; *P*-value = 0.011), gender (COR = 1.61 [1.00–2.57 at 95% CI]; *P*-value = 0.049), ward (COR = 2.02 [1.09–3.72 at 95% CI]; *P*-value = 0.025), marital status (COR = 0.36 [0.13–0.99 at 95% CI]; *P*-value = 0.048), presence of comorbidity (COR = 2.37 [1.46–3.83 at 95% CI]; *P*-value < 0.001), the total number of drugs (COR = 16.49 [9.25–29.38 at 95% CI]; *P*-value < 0.001),and duration since diagnosis (COR = 1.70 [1.02–2.82 at 95% CI]; *P*-value = 0.04), were significantly associated with cs-DDIs at univariate logistic regression. Ten variables including seven that showed statistical significance at the univariate level and three other variables with a *p*-value < 0.25, including admission status, level of education, and previous admission were included in the multivariate analysis (Table 6). In multivariate analysis, only two variables, female gender (aOR = 2.43 [1.23–4.48 at 95% CI]; *P*-value = 0.011)) and concurrent use of ≥ 6 drugs (aOR = 18.82 [9.58–36.95 at 95% CI]; *P*-value < 0.001)) were found to be significantly associated with cs-DDIs (Table 6). Female patients were about 2.43 times more likely (aOR = 2.43: 1.23–4.80 at 95% CI; *p* value = 0.011) to experience a cs-DDI as compared to the males. Patients receiving six or more drugs were about 18.82 times more likely (aOR = 18.82 [9.58, 36.95 at 95% CI]; *P*-value < 0.001) to experience a cs-DDI as compared to those receiving less than six drugs.

**Table 6:** Univariate and Multivariate logistic regression of the factors associated with clinically significant DDIs among cancer patients, Uganda

Independent variables		Univariate logistic regression		Multivariable logistic regression	
Variables	Categories	COR (95% C.I)	*P* value	aOR (95% C.I)	*P* value
Gender^a^	Female	1.61 (1.00–2.57)	**.049**	2.61 (1.37- 4.96)	**.003**
	Male	1		1	
Age in years^a^	< 18	1		1	
	18–59	2.36 (1.22–4.55)	**0.011**	0.01 (0.0–1.1)	0.999
	> = 60	1.54 (.79–2.98)	0.202	0.02 (0–1.3)	0.998
Ward^a^	Adult	2.02 (1.09–3.72)	**.025**	2.01 (.71–5.70)	.188
	Pediatrics	1		1	
Admission status^a^	In Patient	1.75 (.95–3.21)	.071	-	-
	Out Patient	1		-	-
Place of residence	Urban	.88 (.49–1.59)	.676	-	-
	Rural	1		-	-
Marital status^a^	Married	.36 (.13-.99)	**.048**	.51 (.14–1.84)	.300
	Single	.32 (.09–1.13)	.076	.24 (.05–1.18)	.078
	Separated/Divorced/ Widowed	1		1	
Employment status	Unemployed	1.10 (.54–2.23)	.797	-	-
	Employed	1		-	-
Level of education^a^	None	.704 (.35–1.42)	.325	.46 (.19–1.16)	.101
	Primary	.620 (.33–1.17)	.142	.48 (.20–1.15)	.100
	Secondary/Tertiary	1		1	
Previous admission^a^	No	.72 (.45–1.15)	.172	.84 (.41–1.72)	.638
	Yes	1		1	
Previous or concurrent radiation/surgery	No	1.02 (.61–1.70)	.941	-	-
	Yes	1		-	-
Any Comorbidity^a^	No	1		1	
	Yes	2.37 (1.46–3.83)	***P* < .001**	1.47 (.74–2.94)	.272
Total drugs^a^	< 6	1		1	
	≥ 6	16.49 (9.25–29.38)	***P* < .001**	9.14 (4.80–17.43)	***P* < 0.001**
Duration since diagnosis^a^	< 2	1.70 (1.02–2.82)	**.040**	1.07 (.50–2.28)	.855
	> = 2	1		1	
Presence of metastasis	Yes	.96 (.45–2.02)	.907	-	-
	No	1		-	-

*COR* Crude Odds Ratio, *aOR* Adjusted Odds Ratio

Bold: Statistically significant (*p* value < 0.05)

^a^Eligible for Multivariable logistic regression (*P* value < 0.25)

## Discussion

The present study showed that 60.3% of the cancer patients attending MRRHCU experienced at-least one cs-DDI during the study period. This frequency is comparable to 69.73% [17] in Iran among all cancer patients, 67% in the Netherlands [11] among oncology patients from a retrospective cohort study, and another retrospective observational study among hospitalized cancer patients showed a 50% prevalence in Cyprus [18]. Additionally, all the above studies used Lexicomp® drug interaction software like the current study, apart from the study in the Netherlands that used Micromedex drug interactions software which is comparable to Lexicomp® drug interaction software in accuracy [19].

Our study’s cs-DDIs prevalence is considerably higher than some prevalences previously reported among cancer patients. This has been noted from studies reporting 26.8% prevalence among cancer in-patients in the Netherlands [20], and 41% and 46% among cancer out-patients in Spain [21] and the Netherlands [22] respectively. Lower prevalences could be due to considering only one kind/group of patients compared to the current study that considered all patient groups. Secondly, in the Netherlands studies, manual screening methods (peer-reviewed reports) and drug interaction Fact Software version 4.0, 2006 were used to screen for cs-DDIs, which are noted to be less accurate compared to Lexicomp which was used in the current study [19]. This could explain the lower prevalence observed. A clinical trial exclusively among cancer patients in the United States of America revealed that 9.4% of them experienced csDDIs [23]. The restriction to only clinical trial patients and a specific antineoplastic agent could explain the lower prevalence of csDDIs. A low DDI vigilance noted in the low-income state health care settings [24], including oncology units [25], and our study’s inclusion of all cancer patients, may explain the higher csDDI prevalence in the current study. Other possible explanations for this discrepancy include differences in the DDI assessment tool used [20] and differences in the study population [26, 27]. Lower prevalences in previous studies may also be explained by consideration of only antineoplastic interactions excluding interactions between supportive and comorbid medications [21, 23, 26] and considering only risk categories D and X as being clinically significant [21]. Likewise, more than half (58.24%) of the DDIs identified in this study occurred with non-anticancer medications. The most frequently involved antineoplastics were doxorubicin, cyclophosphamide, and methotrexate. These drugs were also reported to be the most frequently involved in previous studies among cancer patients [12, 28]. The high prevalence of cs-DDIs warrants due attention to their prevention among cancer patients as they can lead to unexpected adverse effects and hence a poor prognosis including death [3, 29]. In our study, about three quarters (74.14%) of the cs-DDIs were risk category C that required monitoring, 21.4% were category D required therapy modification and 4.45% were risk category X requiring avoiding therapy, with most of them moderate (71.72%) in severity. The proportion of risk category C cs-DDIs in the current study is comparable to 78.6% from a study in Iran [30]. The proportion of the moderate cs-DDIs in severity observed in our study is comparable with 77% proportion of the moderate cs-DDIs reported among cancer patients in Netherlands [31]. At an Indian teaching hospital, a prospective study among only admitted cancer patients using Drug Interaction Fact software Version 4, revealed a lower prevalence of 56.8% [32]. This may have been due to the exclusion of the out patients and use of a different DDI checker. Another cohort study among breast cancer patients also revealed a lower prevalence of 25.3% [12]. This could be due to considering one cancer group. A 24.2% proportion of moderate to major cs-DDIs reported among clinical trial cancer patients [23] was lower owing to the consideration of only DDIs associated with chemotherapy agents. Being an interventional study design also allowed the investigators to avoid medications with potential DDIs whenever possible. The current high proportion of moderate to major drug-drug interactions among cancer patients requires more emphasis to prevent ADEs due to drug interactions in addition to high ADR risk associated with the antineoplastic agents [12, 33, 34], in the bid to assure patient safety.

In the current study, the use of six and more drugs was significantly associated with clinically significant drug interactions. This finding was comparable with those from previous studies in cervical cancer patients in Pakistan [12, 35] and among cancer patients in the Netherlands [20, 22], all of which reported a higher number of concurrently used medications was significantly associated with cs-DDI. Due to disease sequelae related to cancer and comorbidities, cancer patients are at high risk of polypharmacy [33], and the use of six or more drugs [36, 37]. This predisposes them to cs-DDIs [35, 38].

Being female was shown to be another determinant of the occurrence of clinically significant DDI in the present study. In previous studies, polypharmacy and comorbidities have been noted as prevalent among female neoplasms (Breast cancer and cervical cancer) [39–41]. More studies have shown that comorbidities and polypharmacy were associated with clinically significant DDIs among breast cancer females [12, 42] and cervical cancer patients [41, 43]. This explains the current study’s 87.5% of patients with female-specific neoplasms and 67.7% of patients with breast neoplasms experiencing at least one csDDI; higher than the overall prevalence of 60.3%. This is comparable with the findings of a study among cancer inpatients in Brazil that showed females experience more DDIs than men [44]. Moreover, the higher health-seeking behavior among females [45] in Uganda can explain the higher number of medications and thus, more risk of incurring a clinically significant DDI than in males.

Due to cancer disease and therapy sequelae, for example, pain and ADRs among others, which require pharmacologic intervention, cancer patients are exposed to polypharmacy a noted risk factor of cs-DDIs [20, 35]. This informs the development of medication discrepancy preventive strategies like the involvement of clinical pharmacists in the healthcare management team at the cancer unit, who will reduce csDDIs through conducting medication reconciliation [46]. The development of a verified comprehensive medication reconciliation form and a DDI list at the cancer unit will be so key in detecting, reporting, preventing, and resolving medication discrepancies [47], to mitigate the burden of cs-DDIs and their consequences. Failure to recognize the magnitude of such interactions and implementation of preventive strategies leads to increased health costs, prolonged hospitalization, permanent disability, and sometimes death [48].

### Limitations

The use of one drug-drug interaction checker might limit the comparison with other studies which employed different checkers. We did not include the performance status of the participants as an independent variable in the multivariable logistic regression. Lastly, we did not follow up on the patients with cs-DDIs for occurrences of adverse drug events or treatment failure due to the interaction.

## Conclusion

The current study showed that more than half the patients with cancer attending MRRHCU experienced at least one cs-DDIs, and about three-quarters of these were of category C in risk rating and moderate in severity. Morphine-Metoclopramide was the commonest cs-DDI. Cyclophosphamide-doxorubicin was the most frequent cs-DDI among the anti-cancer agents. Female gender and concurrent use of six or more drugs were found to be significantly associated with cs-DDIs. DDI vigilance at the MRRHCU should be enhanced through the use of DDI lists, DDI checker software, involvement of clinical pharmacists, and increased patient health education in a way of preventing cs-DDIs and thus, mitigating the potential adverse effects. Future researchers should address the potential outcomes of the csDDIs and their correlation with the performance status of cancer patients by using perspective and multi-centered study designs.

## Data Availability

All the data supporting the conclusions of this article are included in this manuscript and any more data needed can be availed by corresponding on request.

## Abbreviations

ADE: Adverse Drug Event
ADR: Adverse Drug Reaction
aOR: Adjusted odds ratio
OR: Odds ratio
cs-DDI: Clinically significant drug-drug interaction
MRRH: Mbarara Regional Referral Hospital
MRRHCU: Mbarara Regional Referral Hospital Cancer Unit
MUST: Mbarara University of Science and Technology
MUST-REC: Mbarara University of Science and Technology Research Ethics Committee
SPSS: Statistical Package for Social Sciences
